# Guanosine fast onset antidepressant-like effects in the olfactory bulbectomy mice model

**DOI:** 10.1038/s41598-020-65300-w

**Published:** 2020-05-21

**Authors:** Roberto Farina de Almeida, Camila Barbosa Pocharski, Ana Lúcia S. Rodrigues, Elaine Elisabetsky, Diogo O. Souza

**Affiliations:** 1 Departamento de Ciências Biológicas, Programa de Pós-Graduação em Ciências Biológicas, Universidade Federal de Ouro Preto (UFOP), Ouro Preto, Brazil; 2 Programa de Pós-Graduação em Ciências Biológicas: Bioquímica, Universidade Federal do Rio Grande do Sul (UFRGS), Porto Alegre, Brazil; 3 Departamento de Bioquímica, Centro de Ciências Biológicas, Universidade Federal de Santa Catarina (UFSC), Florianópolis, Brazil

**Keywords:** Pharmacology, Depression

## Abstract

The treatment of major depressive disorder (MDD) is still a challenge. In the search for novel antidepressants, glutamatergic neuromodulators have been investigated as possible fast-acting antidepressants. Innovative studies suggest that the purine cycle and/or the purinergic signaling can be dysregulated in MDD, and the endogenous nucleoside guanosine has gained attention due to its extracellular effects. This study aimed to verify if guanosine produces fast-onset effects in the well-validated, reliable and sensitive olfactory bulbectomy (OBX) model of depression. The involvement of the mTOR pathway, a key target for the fast-onset effect of ketamine, was also investigated. Results show that a single i.p. injection of guanosine, or ketamine, completely reversed the OBX-induced anhedonic-like behavior 24 or 48 h post treatment, as well as the short-term recognition memory impairment 48 h post treatment. The antidepressant-like effects of guanosine and ketamine were completely abolished by rapamycin. This study shows, for the first time, that guanosine, in a way similar to ketamine, is able to elicit a fast antidepressant response in the OBX model in mice. The results support the notion that guanosine represents a new road for therapeutic improvement in MDD.

## Introduction

Major depressive disorder (MDD), a severe psychiatric condition, is characterized by high prevalence^1^ and disability for affected individuals^2,3^, leading to elevated economic burden^1,4^ and a substantial percentage (circa 30%) of patients unresponsive to treatment^4,5^. Though this picture vindicates the search for superior medication^6^, unfortunately most research results has been limited to more drugs with the same mechanism of action, or at best certain incremental innovation^7,8^.

A major flaw in treating depression is the time lag between the onset of the treatment and the remission of the symptoms^4^. Especially relevant for treating patients is skepticism, this delay obstructs adhesion, prolongs suffering and disability, and increase suicide risk^4,9^. Ketamine has emerged as a fast-acting antidepressant agent that is effective to elicit rapid effect even for severe depressed patients refractory to antidepressant treatment^10^. However, ketamine seems to be an unsecure drug especially under repeated administration. This limitation of ketamine’s use has lead to renewed efforts for pursuing novel fast-acting antidepressant agents, especially glutamatergic neuromodulators, including guanosine (GUO)^9,11–13^. Several lines of evidence suggest that the purine cycle and/or the purinergic signaling could be dysregulated in MDD patients^14–17^. As purinergic signaling modulate cell proliferation, differentiation, neuron-glia crosstalk, and inflammation^17–19^, disturbances in purine homeostasis contribute to the pathophysiological process underlying MDD^15,18^. GUO is an endogenous nucleoside with neuroprotective effects shown in different animal models of brain disorders^17,20,21^. In conditions under which brain homeostasis is disrupted GUO seems to play an important role as an extracellular signaling molecule, modulating neurotrophins, displaying antioxidant activity, attenuating inflammatory response and glutamatergic toxicity^17,21^. In regard to depression, while preclinical studies showed that GUO produces antidepressant-like effects in animals models with predictive validity^11,22,23^, it is of particular interest that a clinical and longitudinal study showed decreased GUO serum levels in MDD patients in comparison with non-depressed controls^14^.

Among the rodent models that can predict onset delays of classical (weeks) and fast-acting (hours-days) antidepressants, the olfactory bulbectomy (OBX) and the unpredictable chronic mild stress (UCMS) stand out^9,24,25^. As reliability and practical use are vital aspects of animal models for translational research, OBX presents advantages over the UCMS^9^. Considering the interest in novel and fast-acting antidepressant agents, the purposes of this study were: (1) to investigate if systemic GUO induces fast-onset antidepressant effects in the OBX model of depression in mice, and (2) verify the involvement of the mechanistic target of rapamycin (RAP), the mammalian target of rapamycin (mTOR) pathway, a key target in the fast onset effect of ketamine (Ket)^12^.

## Results

**Figure 1:**
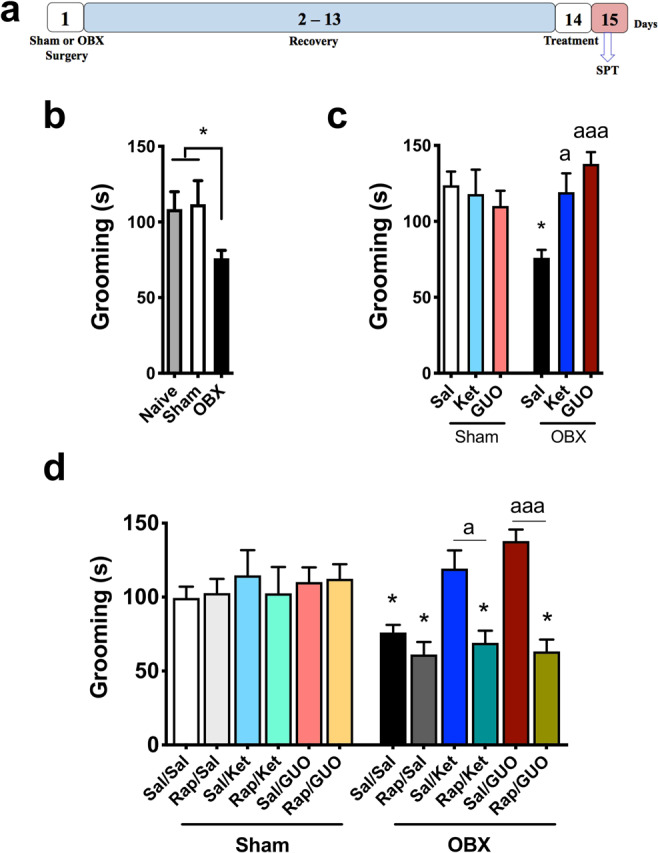
Effects of OBX, ketamine (Ket), guanosine (GUO) and pre-treatment with rapamycin (RAP) on the splash test (SPT). (**a**) experimental design; (**b**) compares naïve, Sham and OBX groups; (**c**) effects of Ket and GUO in Sham and OBX mice; (**d**) effects of RAP pre-treatment on Sham and OBX mice treated with Ket or GUO. One-way ANOVA/Tukey´s was used to compare the Naïve, Sham and OBX groups (n = 8–20). The effects of treatment (Ket or GUO, n = 15–20) were compared by Two-way ANOVA/Tukey. The effects of RAP pre-treatment (n = 12–20) were compared by Three-way ANOVA/Tukey. Columns represent mean ± S.E.M. *p < 0.05 compared to the respective Sham group; ^a^p < 0.05, and ^aaa^p < 0.001 compared to KET and GUO groups (respectively) without Rap pre-treatment.

Figure 1 shows the effects of GUO and Ket in the Splash test (SPT). Figure 1a. illustrates the experimental design. One-way ANOVA reveals a significant difference (F(_2,33_) = 5.46, P = 0.008; Fig. 1b), while Tukey shows that only OBX mice presented decreased grooming (P = 0.032 and P = 0.029; 1b). Two-way ANOVA detects a treatment effect (Fig. 1c; F(_2,99_) = 3.163; P = 0.046) and interaction between treatments (Ket and GUO) and condition (Sham or OBX) (F(_2,99_) = 5.44; P = 0.006; 1c). Tukey indicates that Ket or GUO administered twenty-four hours before the SPT completely reversed the effect of OBX in grooming (P = 0.011, P = 0.019, P < 0.001; 1c). Three-way ANOVA shows a significant treatment effect (F(_2,189_) = 3.94; P = 0.021; Fig. 1d). Additionally, there were interactions between treatment and condition (F(_1,189_) = 7.35; P = 0.007; Fig. 1d), and RAP pre-treatment and treatments (Fig. 1d; F(_1,189_) = 11.39; P = 0.0007; Fig. 1d). Tukey points that RAP pre-treatment completely abolished the antidepressant-like effect of Ket and GUO (P = 0.01, P < 0.0001; 1d), and indicates significant effects of OBX (P = 0.044, P = 0.0243, P = 0.048, P = 0.033; 1d).

**Figure 2:**
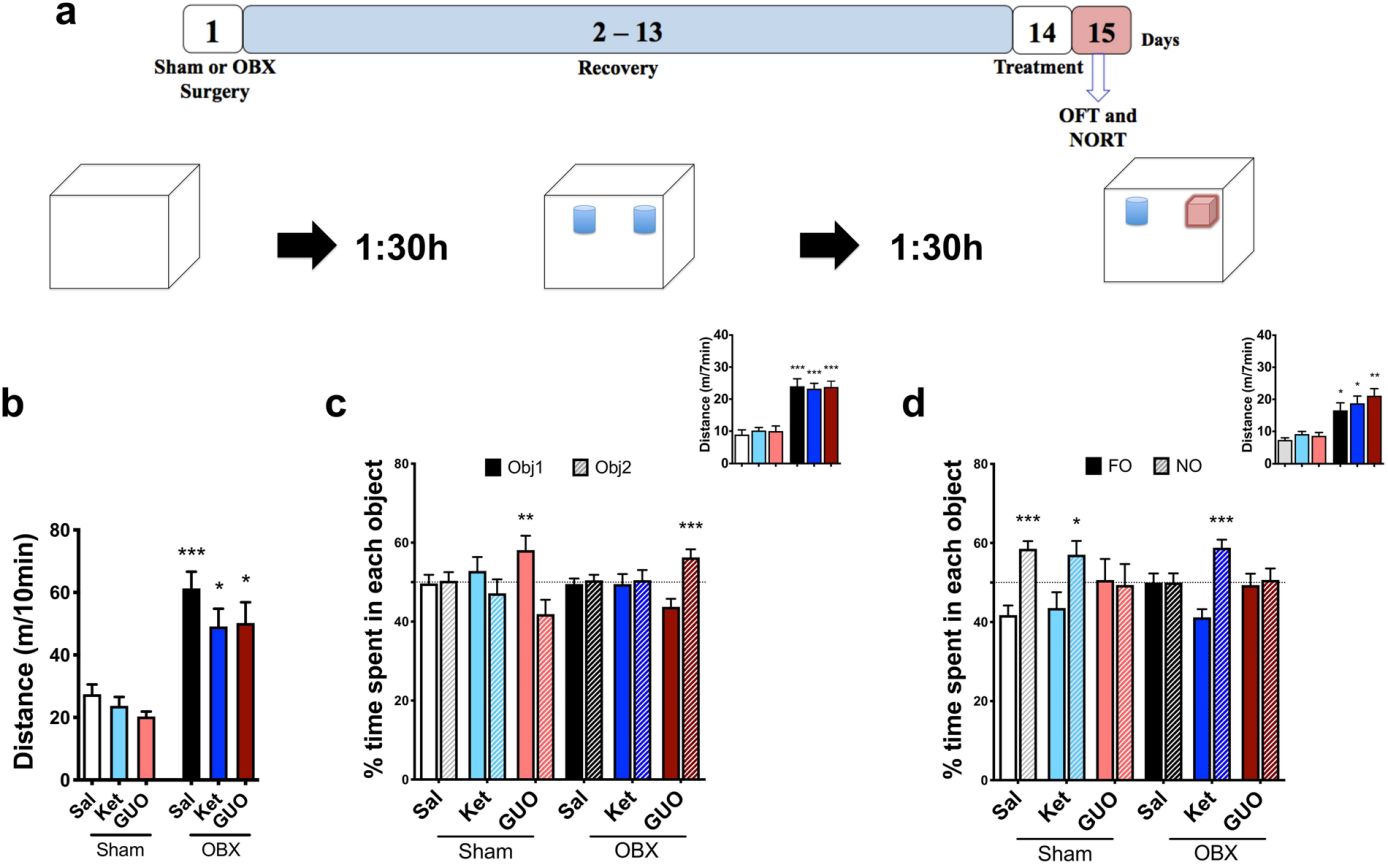
Effects of ketamine (Ket) and guanosine (GUO) in Sham and OBX mice in the open field (OFT) and novel object recognition test (NORT). (**a**) experimental design; OFT was conducted 90 min before NORT training, and test performed 90 min post training; OFT and NORT were performed 24 h after treatments; (**b**) effects of Ket and GUO in the OFT; effects of KET and GUO in NORT training (**c**) and test (**d**) sessions. Inserts represent locomotor activity. FO represents familiar objects and NO represents Novel Object Columns represent mean ± S.E.M. n = 10–12 animals per group. Distance travelled were compared by two-way ANOVA/Tukey. The effects of treatments in NORT were analyzed by two-way ANOVA/Sidak. * p < 0.05, ** p < 0.01 and ***p < 0.001 compared to the respective Sham group.

Figure 2 shows the effects of OFT and NORT performed 24 hours after treatments. The experimental design is illustrated in Fig. 2a. Two-way ANOVA reveals a significant effect of OBX in ambulation (F(_1,66_) = 40.00, P < 0.0001; Fig. 2b). Tukey reveals that neither Ket nor GUO altered the hyperactivity of OBX (Fig. 2b; P < 0.0002, P = 0.026, P = 0.015; 2b). The hyperactivity of OBX mice persisted in NORT training and test session (Fig. 2c and d inserts; F(_1,66_) = 82.79, P < 0.0001 and F(_1,66_) = 39.72, P < 0.0001, respectively). Tukey indicates that treatments were not able to reverse the increased distance travelled by OBX mice in NORT training and test sessions (P < 0.0001 [Fig. 2c insert] and P = 0.014, P = 0.012, P = 0.001 [Fig. 2d insert], respectively). Two-way ANOVA shows lack of effects from treatment or condition in NORT training session (Fig. 2c; F(_2,132_) = 0.63, P = 0.5345 and F(_1,132_) = 2.74, P = 0.1018). Sidak identifies an increased time spent exploring one of the two objects in Sham and OBX groups treated with GUO (objects 1 and 2, respectively, P = 0.003 and P = 0.0004; 2c). Two-way ANOVA reveals an OBX main amnesic effect in NORT test session (Fig. 2d; F(_2,132_) = 3.52; P = 0.0351). Sidak shows that the OBX- induced memory impairment was reversed by Ket (but not GUO) (P < 0.0001; 2d), while in Sham groups mice treated with Sal and Ket (but not GUO) presented intact recognition memory (P = 0.0002 and P = 0.0359; 2d).

Twenty-four hours after NORT the same mice were exposed to the SPT. In Supplementary Fig. 1b Two-way ANOVA indicates a main effect of treatments (F(_2,66_) = 7.65, P = 0.0010) and interaction between condition and treatment (F(_2,66_) = 3.57, P = 0.033). Tukey identifies that Sham is different from OBX (*P = 0.028), and that Ket and GUO (P = 0.0031 and P = 0.0003, respectively) are different from saline (Supplementary Fig. 1b).

**Figure 3:**
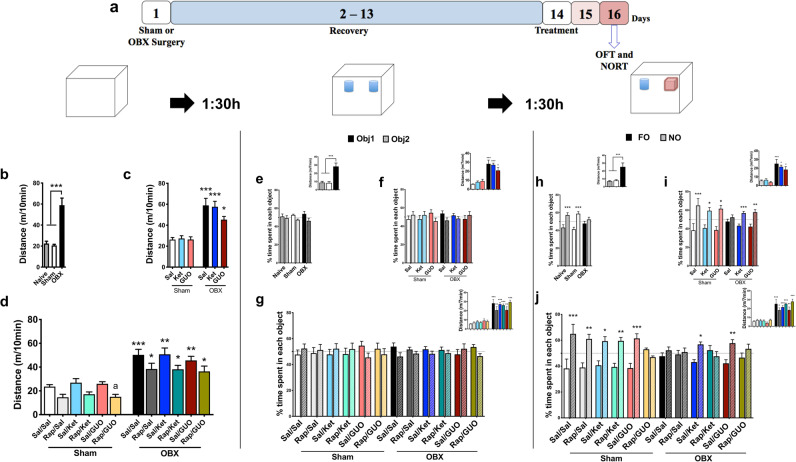
Effects of ketamine (Ket) and guanosine (GUO), and pretreatment with rapamycin (RAP), on the open field (OFT) and novel object recognition test (NORT) in Sham and OBX mice. OFT and NORT were performed 48 h after treatments. (**a)** experimental design; OFT was conducted 90 min before NORT training, and test performed 90 min post training. Left column: OFT: distance travelled by naïve, Sham and OBX groups (**b**), effects of Ket and GUO (**c)**, effects of RAP pretreatment (**d**). Middle column: NORT training: naïve, Sham and OBX groups **(e**), effects of Ket or GUO (**f**), effects of RAP pre-treatment (**g**) groups. Left column: NORT test: naïve, Sham and OBX groups (h), effects of Ket or GUO (i), effects of RAP pre-treatment (j). Inserts represent locomotor activity. One-way ANOVA/Tukey´s was used to compare the Naïve, Sham and OBX groups (n = 8–20). FO = familiar object; NO = novel object Distance travelled by Sham or OBX groups in OFT and NORT were analyzed by two-way ANOVA/Tukey. Data with RAP pre-treatment were analyzed by three-way ANOVA/Tukey for distance travelled and three-way ANOVA/Sidak for NORT, n = 12–20. Columns represent mean ± S.E.M. * p < 0.05, * p < 0.01 and ***p < 0.001 compared to the respective Sham group.

Figure 3 shows the results of OFT and NORT performed 48 hours after Ket and GUO, as well as the effects of RAP pre-treatment. The experimental design is illustrated in Fig. 3a.

One-way ANOVA reveals a main effect of the OBX in the total distance travelled in OFT (F(_2,33_) = 12.62, P < 0.0001; 3b), NORT training (F(_2,33_) = 14.86, P = 0.0002; 3e insert) and test (F(_2,33_) = 11.50, P = 0.0005; 3 h insert) sessions. Tukey confirms differences as indicated by figure asterisks. Two-way ANOVA indicate main effect of condition regarding distance travelled in the OFT (F(_1,75_) = 56.40; P < 0.0001; 3c), NORT training (F(_1,75_) = 57.94, P < 0.0001; 3 f insert) and test (F(_1,75_) = 43.33, P < 0.0001; 3i insert) sessions. Three-way ANOVA reveals the main effect of condition in locomotion (F(_1,135_) = 62.80; P < 0.0001; 1d) regardless of RAP pre-treatment in the OFT (F(_1,135_) = 13.73; P = 0.0008; 1d), NORT training (F(_1,135_) = 144.4, P < 0.0001; 3 g insert) and test (F(_1,135_) = 143.0; P < 0.0001; 3j) sessions. Tukey’s post hoc test corroborates the differences as indicated by asterisks.

Concerning the object recognition, one-way ANOVA shows that there were no differences in NORT training performance regardless of condition (F(_2,33_) = 0.29, P = 0.743; 3e) and a main effect of OBX in test performance (F(_2,33_) = 6.31, P = 0.006; 3 h). Tukey indicates that recognition memory was in place and identifies that only OBX mice present memory deficit (P = 0.0016 and P = 0.0002; 3 h). In NORT training session, two-way ANOVA reveals any significant difference in time spent in each object (Fig. 3f). Two-way ANOVA shows a significant condition effect in NORT test session (Fig. 3i; F(_1,150_) = 4.05, P = 0.048) with no significant interactions (F(_5,150_) = 2.04; P = 0.076; 3i). Sidak shows that all Sham groups increased the time spent in exploring the NO at test session (P < 0.0001, P = 0.022, P = 0.0006; 3i), while OBX-induced memory deficit was completely reversed by Ket and GUO (P = 0.046 and P = 0.008, respectively; 3i).

In another cohort of animals, the effect of RAP pre-treatment was investigated. At NORT training session, no main effect was detected in object recognition (F(_2,135_) = 0.79; P = 0.457; 3 g), while at NORT test three-way ANOVA reveals that OBX model (Fig. 3j; F(_1,135_) = 6.25; *P = 0.0138) and RAP pre-treatment (Fig. 3j; F(_1,135_) = 5.03; P = 0.0268) presented a significant interaction. Sidak identifies that RAP pre-treatment completely blocked the memory protection afforded by Ket and GUO, while inducing memory deficit in the Sham GUO treated group (P = 0.0001, P = 0.009, P = 0.015, P = 0.038, P = 0.009, P = 0.0002 and P = 0.004, respectively; 3j).

## Discussion

Endogenous glutamatergic neuromodulators have been proposed to elicit fast antidepressant responses, possibly sharing with Ket common neurobiological mechanisms^26^. We provide robust evidence that a single i.p. injection of the nucleoside GUO (7.5 mg/kg) completely reverses the anhedonic-like behavior (within 24 h) and memory impairment (within 48 h) induced by OBX, a well validated, reliable and sensitive MDD rodent model. The effects of GUO were comparable to those elicited by Ket (10 mg/kg), used as positive control. The antidepressant-like effects of GUO and Ket were completely blocked by pre-administration of  RAP, suggesting the involvement of the mTOR pathway.

We showed that the anhedonic-like behavior (decreased grooming behavior) observed in the OBX mice model is a transient effect that persists up to 4 weeks after the bilaterally removal of olfactory bulbs, whereas the hyperactivity (assessed by OFT) persist up to 8 weeks^27^. The fast effect of Ket in the OBX model is remarkably compatible with clinical data^10,28^ adding to the translational value of OBX. Our data replicates the findings of Holubova *et al*. (2016) for Ket in the same model, though different experimental design. In this study it is clear that the diminished self-care and motivational behavioral induced by OBX was completely reversed 24 h after a single adminitration of GUO. The potential antidepressant-like effects of acute GUO was previously suggested by using rodent screening tests, as the forced swimming, tail suspension and/or acute restraint stress tests^23,29^. However, this is the first study to show that GUO acts as fast onset antidepressant in a rodent model with translational value that can accurately predict time of onset to antidepressant effect^9,25^. In this context, it is noteworthy that a single administration of GUO and Ket resulted in comparable fast-onset antidepressant effects under the same experimental design and laboratory conditions.

When NORT was performed twenty-four hours after treatments, the OBX-induced recognition memory deficit was reversed by Ket but not GUO. Though the widely distributed NMDA receptors^30^ are required for some forms of long-term potentiation (LTP)^31^, under certain conditions its antagonism can, paradoxically, improve learning and memory process^32^. This is the case with memantine, for which meaningful enhancement in memory features, including LTP, is reported^33,34^. Though Ket acts as a noncompetitive NMDAr antagonist^13,35^, the reversal of memory impairment by Ket in the OBX model is in agreement with the fact that Ket infusions were associated with improvement in learning, partly accounting for amelioration in depression symptoms over time^36^. The lack of effects of GUO on memory deficit within 24 h might be associated with the well-documented amnesic effect of GUO in rodents^37–41^. Amnesic effects of GUO in (7.5 mg/kg, i.p. administered before training session) inhibitory avoidance acquisition (24 hours post training) were observed by Roesler *et al*.^41^, Saute *et al*. ^40^ and Giuliani *et al*.^37^ in rats, by Vinadé and colleagues in rats and mice^38^, and in spontaneous alternation in mice by Tort and collaborators^39^. We here extend this finding to the object recognition test with Sham and OBX mice treated with where GUO (7.5 mg/kg) consistently induced impaired performance in test session. GUO amnesic effects are likely to be associated with adenosine A1 receptor agonism^42–44^, with consequent inhibition of glutamate release and disruption of learning and memory^45,46^. Postulating that the amnesic effect of GUO is transient, another cohort of animals were submitted to NORT 48 h after treatment with GUO or Ket, when the memory deficit was reversed by both. The reversal of memory deficits in olfactory bulbectomized mice by GUO is another novelty of this study, and is in line with MDD pharmacotherapy demands^47^. Of relevance for GUO clinical usefulness, several studies indicate that chronic GUO administration does not present meaningful toxicities^17,20,21^. If the results with GUO in OBX mice is replicated in clinic, the GUO-induced transient amnesic effect may constitute a minor side effect, especially in comparison to the psychotomimetic effects of Ket that limit its clinical usefulness^48^.

Ket and GUO antidepressant effects in OBX mice were completely abolished by RAP pre-treatment. This result corroborates the idea that the mechanism of action of Ket as fast-onset antidepressant involves the activation of mTOR signaling pathway and the consequent phosphorylation of the p70 ribosomal S6 protein kinases (p70S6K)^12^. The participation of mTOR pathway in GUO effects in OBX mice is consistent with the reported activation of the mTOR/PI3K/Akt pathway for the antidepressant-like effects of GUO in other rodent models to study MDD^11,22^. The activation of this pathway facilitates protein translation, cell growth and proliferation pathways, especially of proteins required for the formation, maturation, and function of new spine synapses^49^. Several *in vitro* and *in vivo* studies demonstrate that GUO increases the proliferation of neuroprogenitors cell, suggesting that increased neurogenesis may be in place^29,50,51^ as also reported for Ket antidepressant mechanism of action^12,13,52^. Though GUO is considered an orphan neuromodulator^20^, this study adds evidence to its extracellular effect and a ‘downstream’ signaling pathway.

Neither Ket nor GUO affected the hyperactivity induced by OBX, a hallmark of the OBX behavioral phenotype^9,53^. Magnetic resonance studies showed that OBX leads to gross structural changes in rodent brain including significant amygdala impairment^54,55^. The amygdala, involved in the emotional salience of environmental stimuli and modulation of affective states^56^, seems to be key for OBX hyperactivity. Contrasting neurogenesis mechanisms modulate the hippocampus and prefrontal cortex functionality in comparison with the amygdala in rodents submitted to different MDD or stress models^57^. Different modulatory mechanisms are consistent with GUO and Ket effects in SPT and NORT despite the presence of hyperlocomotion. The fact that both drugs were able to reverse OBX-induced transient behavioral disturbances, but not hyperactivity, speaks of a specific effect of these drugs in the neuronal circuitry relevant to depression.

To the best of our knowledge this is the first pre-clinical demonstration that GUO acts as fast-onset antidepressant in an animal model that mimics some aspects of depressive symptoms seen in humans. The reversal of antidepressant-like effects by rapamycin in the OBX model reinforces the notion that the mTOR pathway mediates Ket antidepressant effects, and, most importantly, that this mechanism is shared with GUO. This study reinforces the potential antidepressant effect of GUO and, given that purine cycle dysregulation can be of relevance in the course of MDD, hopefully opens news perspective for therapeutic developments.

## Materials and Methods

### Animals

Six cohorts of seven-weeks-old male C57BL/6 (25 g) mice, were kept under a 12-h light/dark cycle (light on at 7:00 AM) at 22  ±  1 °C in polypropylene cages (30 × 20 × 13 cm; 5 per cage) with water and food available *ad libitum*. Twenty-four or forty-eight hours before the behavioral test, animals were taken to the behavioral room with appropriate lighting (200 lx) to acclimatize (at least 2 hours) with the new environment. After this habituation period, in the same room, mice of different cohorts were randomly divided into the different groups and treatments schedules. Behavioral experiments were carried out between 1-6 PM. Experimental procedures complied with official National Council for Animal Experimentation Control (CONCEA – Brazil) for the care and use of laboratory animals and were approved by the Ethical Committee of the Federal University of Rio Grande do Sul (Project approval #30124). The animals were maintained according to the National Institutes of Health Guide for the Care and Use of Laboratory Animals (NIH Publications No. 8023, revised 1978).

### Bilateral olfactory bulbectomy

The bilateral OBX was performed as previously described^27^. Mice were anaesthetized with (i.p.) xylazine (6 mg/kg) and ketamine (100 mg/kg) diluted in saline. After placed in the stereotaxic apparatus the head was shaved and the scalp incision made above the olfactory bulbs (4 mm rostral to bregma). A burr hole (circa 2 mm in diameter) was made and the olfactory bulbs removed with surgical micro scissors and suction with a glass Pasteur pipette. After suturing the incision, animals were conducted to the postoperative period of 14 days. OBX is routinely performed without analgesic or anti-inflammatory drugs^27,58,59^. At the end of the experiment animals were sacrificed and the cerebral cortex meticulously analyzed. Animals presenting incomplete bulbs removal (<2/3) and/or injured frontal cortex were excluded from the study. About 2–5% of mice per experiment were excluded from the statistical analysis due to surgical complications.

The model was validated by comparing naïve, sham and OBX mice. Naïve and sham groups were compared to exclude experimental bias associated with the surgical procedure. Figure 1(b) and 3(b,e,h), illustrate these three groups in the distinct behavioral parameters.

### Drugs

Guanosine (GUO) and rapamycin (RAP) were purchased from Sigma Chemicals (St. Louis, MO, USA). Ketamine was obtained from Rhobifarma Indústria Farmacêutica Ltda (Hortolândia, SP, Brazil). All drugs were dissolved in saline (0.9% NaCl) except RAP that was dissolved in saline plus 20% of DMSO as previously determined^60^.

Treatments were applied intraperitoneally (10 ml/kg). Saline, Ket (10 mg/kg) or GUO (7.5 mg/kg) were administered 24 or 48 hours before behavioral experiments; RAP (1 mg/kg) was injected 30 min prior to Saline, Ket or GUO.

### Behavioral protocols

#### Splash test (SPT)

The splash test was used to evaluate self-care and motivational behavior^27^. A 10% sucrose solution was sprayed (0.2 mL/spray) 3 times on the dorsal coat of mice. As the sucrose solution soils the mouse fur subjects initiate grooming: a decrease in grooming reflects loss of self-care and motivation, which are strongly related to anhedonia. The time spent on grooming behaviors during the first 5 min after application of the sucrose solution was recorded. The apparatus was cleaned with 70% alcohol and dried after each test. Grooming behaviors, including licking, scratching and/or face-washing, were analyzed by experienced researcher blind-to-treatment for each mouse individually.

#### Open field test (OFT)

The OFT was run as previously described^27^, in order to investigate locomotor/exploratory activity; hyperactivity is one of the earliest and most accepted indices (gold standard phenotype) of behavior in OBX rodents. Mice were individually placed near the sidewall of a gray wooden box (30 × 30 × 30 cm) with a 200 lx white light and recorded for 10 min by a video-camera positioned above and at ca. 90° to the square arena. The apparatus was cleaned with 70% alcohol and dried after each test. The videos were analyzed by the AnyMaze software (Stoelting Co., Wood Dale, IL).

#### Novel object recognition task (NORT)

The NOR test was carried out as previously reported^61^, with minor modifications, to evaluate recognition memory. The OFT (as above) was performed before NOR in order to reduce the novelty effect of the arena and decrease anxiety levels. Ninety minutes after the OFT mice were submitted to the NORT training session: mice were individually placed at the NORT arena (30 × 30 × 30 cm) containing two identical objects located at opposite and symmetrical corners of the arena, and allowed to explore for 7 min. To prevent coercion to explore the objects mice were placed at the arena facing the wall opposite to the objects. During the training session both objects are novel and the time spent on both objects is expected to be similar (subjects with training session exploration time inferior to 20 s were excluded from the experiment [circa 10%]^62^). Ninety minutes after the training session the test session was performed: mice were individually placed back in the arena with one familiar object (FO, same as in training session) and one novel object (NO) and recorded for 7 min. Videos from the training and test sessions were analyzed by an experienced observer blinded to groups and treatments. Exploration episodes were considered only when the animal’s nose or mouth was in contact with the objects. If recognition memory is present a longer time spent in exploring the NO over the FO is expected.

### Statistics

The Shapiro-Wilk normality test was used to verify gaussian distribution. One-way followed by Tukey´s multiple comparison test was used to compare naïve, Sham and OBX animals in behavioral tests. The distance travelled by Sham or OBX groups treated or not with Ket and GUO in the OFT and NORT, as well as the time of grooming behavior in the SPT were analyzed by two-way ANOVA followed by Tukey’s multiple comparisons test. The effects of treatments in NORT training and test sessions in Sham or OBX groups were analyzed by Two-way ANOVA followed by Sidak’s multiple comparisons test. A three way-ANOVA (mixed-effects model - REML) was used to test the effects of RAP pre-treatment in the Ket or GUO effects in Sham or OBX mice in SPT grooming, OFT and NORT distance travelled, and in NORT object recognition, using the following factors: condition (Sham or OBX); treatments (Sal, Ket or GUO) and RAP pre-treatment; Tukey´s (SPT, OFT and NORT-inserts) or Sidak’s (FO and NO exploration) multiple comparison post-hoc test were used. All statistical procedures and artwork were carried out with Graph Pad Prism (Graph Pad Software, version 8, San Diego, CA, USA). Differences were considered statistically significant at *p*  <  0.05.

### Compliance with ethical standards

All experiments followed the guidelines of the National Institutes of Health Guide for Care and Use of Laboratory Animals (NIH Publications No. 8023, revised 1978). Experimental procedures complied with official National Council for Animal Experimentation Control (CONCEA – Brazil) for the care and use of laboratory animals and were approved by the Ethical Committee of the Federal University of Rio Grande do Sul (Project approval #30124).

## Supplementary information

Supplementary Figure

## Data Availability

Authors declare that all data will be available upon any request.
