# Supplementary figures and images for: Guanosine fast onset antidepressant-like effects in the olfactory bulbectomy mice model
Source: Sci Rep. 2020 May 21;10:8429. doi: 10.1038/s41598-020-65300-w (PMC7242421; doi:10.1038/s41598-020-65300-w)

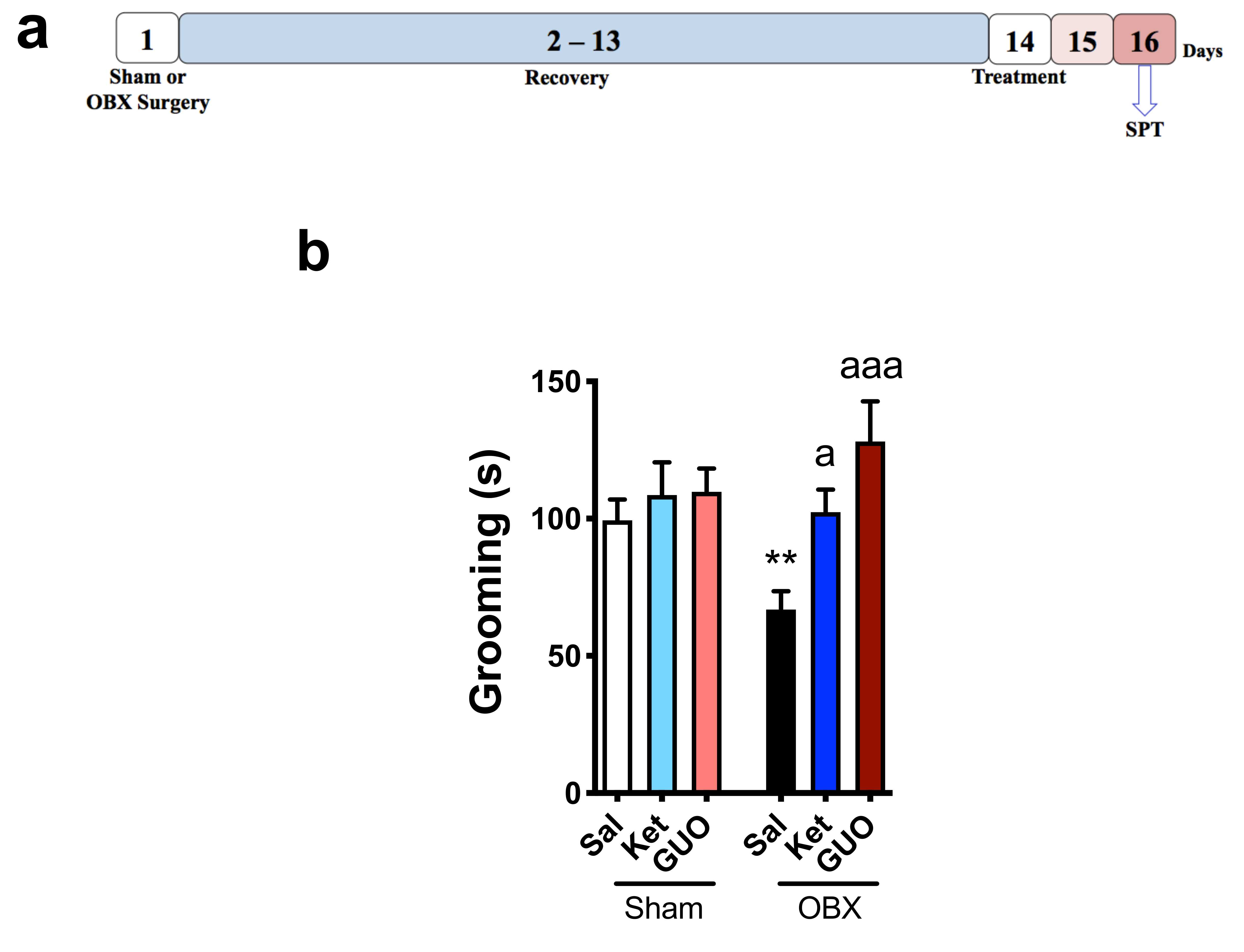

Supplement: Supplementary file 1 — Supplementary Figure [file 41598_2020_65300_MOESM1_ESM.jpg]
